# Interventions to Ensure the Continuum of Care for Hypertension During the COVID-19 Pandemic in Five Indian States—India Hypertension Control Initiative

**DOI:** 10.5334/gh.1010

**Published:** 2021-12-08

**Authors:** Abhishek Kunwar, Kiran Durgad, Prabhdeep Kaur, Meenakshi Sharma, Leimapokpam Swasticharan, Madhavi Mallela, Ashish Saxena, Sadhana Tayade, Sandeep Gill, Bipin K. Gopal, Anupam K. Pathni, Fikru T. Tullu, R. S. Dhaliwal, Balram Bhargava

**Affiliations:** 1 WHO Country Office for India, New Delhi, IN; 2 ICMR-National Institute of Epidemiology, Chennai, IN; 3 Indian Council of Medical Research (ICMR), New Delhi, IN; 4 Directorate General of Health Services, Ministry and Health, and Family Welfare, New Delhi, IN; 5 State NCD Cell, Department of Health, Medical and Family Welfare, Govt of Telangana, Hyderabad, IN; 6 State NCD Cell, Directorate of Health Services, Govt of Madhya Pradesh, Bhopal, IN; 7 State NCD Cell, Directorate of Health Services, Govt of Maharashtra, Mumbai, IN; 8 State NCD Cell, Department of Health and Family Welfare, Govt of Punjab, Chandigarh, IN; 9 State NCD Cell, Department of Health and Family Welfare, Govt of Kerala, Trivandrum, IN; 10 Resolve to Save Lives, New Delhi, IN

**Keywords:** Hypertension, India Hypertension Control Initiative, Community drug distribution, COVID-19 pandemic

## Abstract

**Background:**

Hypertension is the leading risk factor for cardiovascular disease in India, but less than 10% of the estimated people with hypertension have blood pressure under control. The India Hypertension Control Initiative (IHCI) was implemented to strengthen hypertension management and control in public sector health facilities. Since late March 2020, lockdown due to the COVID-19 pandemic limited healthcare access and disrupted the provision of essential health services. IHCI quickly implemented adaptive interventions to improve access to medications.

**Objectives:**

To estimate the availability of antihypertensive drugs in peripheral public sector facilities during the lockdown and the proportion of patients who received drugs through community drug distribution, i.e., through Health and Wellness Centers (HWCs)/Sub-Centers (SCs), the most peripheral public sector health facilities for primary care, and home delivery.

**Methods:**

We collected data from 29 IHCI districts of 5 states (Kerala, Madhya Pradesh, Maharashtra, Punjab, and Telangana) during April–May 2020. The population included individuals diagnosed with hypertension and enrolled under IHCI in all public sector primary care health facilities. We contacted a convenience sample of more than one-third of the functional HWC/SC and analyzed the proportion of facilities and patients who received drugs. We also contacted a convenience sample of patients telephonically to estimate their self-reported availability of drugs.

**Conclusion:**

Of the 4245 HWC/SC, more than one-third were contacted telephonically, and 85–88% had received antihypertensive medications for community-level distribution. Among 721,675 patients registered until March 2020, 38.4% had received drug refills through HWC/SC or home delivery by frontline workers during the lockdown. We demonstrated the feasibility of community-level drug distribution for patients with hypertension during the COVID-19 lockdown in India. The adaptive strategy of community-based drug distribution through HWC/SC and home delivery appears feasible and may help improve access to hypertension care during the COVID-19 pandemic and beyond.

## 1. Introduction

Over 200 million Indian adults live with high blood pressure in India, and less than 10% of them have it under control [1,2]. The India Hypertension Control Initiative (IHCI), launched in November 2017, aims to reduce premature cardiovascular deaths by strengthening hypertension management and control at primary and secondary public health care facilities. The initiative includes a standard hypertension control program approach drawn from the World Health Organization (WHO) HEARTS technical package for cardiovascular disease management in primary health care [3]: a simple, standardized hypertension treatment protocol; regular and uninterrupted supply of medications and validated blood pressure monitors; team-based care and task sharing; patient-centered care; and information systems that allow continuous real-time monitoring. By March 2020, the IHCI had enrolled 721,675 patients with hypertension in 29 districts across five states (Kerala, Madhya Pradesh, Maharashtra, Punjab, and Telangana).

The WHO declared coronavirus disease 2019 (COVID-19), caused by Severe Acute Respiratory Syndrome Coronavirus 2 (SARS-CoV-2), a pandemic in March 2020. To combat the spread of COVID-19 in India, a nationwide mobility restriction, or ‘lockdown,’ was declared from March 24 until the end of May. After that, various state governments relaxed the restrictions gradually. The COVID-19 restrictions not only limited access to healthcare due to severe travel limitations but also disrupted the provision of essential public health services due to the closure of routine out-patient services, re-purposing of health care staff for COVID-19 related duties, and disruption in the transportation of essential medicines and supplies.

Reduced access to essential services during the COVID-19 period is neither unique to hypertension care nor India. Reductions in access to Human Immuno-deficiency Virus (HIV), Tuberculosis (TB), family planning, immunization, and other health services have been documented across multiple regions [4,5]. Programs are adapting to mitigate the impact of COVID-related restrictions. Still, there is a dearth of evidence to inform which interventions are most beneficial to maintain continuity of care, especially for non-communicable diseases (NCDs). The National Programme for Prevention and Control of Cancer, Diabetes, Cardiovascular Diseases, and Stroke (NPCDCS) aims to provide screening and treatment for NCD in India. There were disruptions in the healthcare delivery due to lockdown. Therefore, NPCDCS issued new guidelines to enable continuity of care for patients with hypertension [6].

Antihypertensive drugs are routinely dispensed at Primary Health Centers and other higher facilities such as Community Health Centers and District hospitals. Because of COVID-19 restrictions and lockdown, ‘community-level drug distribution’ was done through Health & Wellness Centers, Sub centers, and at doorsteps by frontline health workers.

We assessed the extent of implementation of community-level distribution of antihypertensive drugs, which was one of the mitigation strategies. We estimated the proportion of Health and Wellness Centers (HWCs) and Sub Centers (SCs) that received antihypertensive drugs and the proportion of patients who had access to medications during the lockdown through community-based drugs distribution in 29 districts across five IHCI project states in India.

## 2. Methods

### 2.1. Design and Setting

We collected data from 29 districts of 5 states (Kerala, Madhya Pradesh, Maharashtra, Punjab, and Telangana). The population included all individuals diagnosed with hypertension and enrolled for hypertension treatment in 5,668 public sector health facilities—88 District/Sub-district Hospitals, 222 Community Health Centers (CHCs), 1113 Rural/Urban Primary Health Centers (PHC), and 4,245 HWCs and SCs.

Before COVID-19, patients with hypertension were registered and provided one-month medications at the PHCs in rural areas and District/Sub-district hospitals/PHCs in urban areas. District hospitals cater to the entire district for secondary care. CHCs cater to around 100,000 population whereas PHCs cater to 30,000 population and have 5–6 SCs (each catering to approximately 5,000 population) for field-level public health activities. In recent years, a national-level government initiative upgraded many subcenters to HWCs, with a dedicated paramedical health staff with advanced training, namely Community Health Officers (CHOs). They are involved in screening for hypertension and diabetes under the nation’s population-based screening program. Most states are gradually operationalizing HWCs in a phased manner in most states. However, the COVID-19 pandemic provided an opportunity to accelerate the utilization of HWCs for drug dispensing and community-level drug distribution. CHOs were involved in delivering refills for patients already prescribed medications.

### 2.2. Description of the intervention

Under IHCI, drug procurement is facilitated by improved forecasting and procurement practices using the state-specific standard treatment protocol. To ensure patient-centered care, most public health facilities dispensed a one-month supply of drugs to all patients with hypertension at the PHC level and above in the implementing districts. Before COVID-19, drug refills were mostly provided at PHC or higher-level facilities, often located far from patients’ homes. Drugs were not routinely distributed at the community level, and HWCs/SCs were slowly being operationalized to dispense medicines with limited utilization.

During COVID-19, based on expert inputs, the Ministry of Health & Family Welfare, Government of India, gave recommendations for extended (up to three-month) prescriptions and community-level distribution of drugs to patients with NCDs, which was absent before COVID-19. IHCI districts implemented critical mitigation interventions, including community-level distribution of antihypertensive medications and drug refills at HWCs or SCs and dispensing medications for extended periods to patients with hypertension.

During the lockdown, all the protocol drugs were mobilized to HWCs and SCs in all IHCI districts, as per the requirement calculated, using a patient line list. At the village level, HWCs and SCs provided drug refills close to patients’ homes with medications for at least 30 days [7]. Besides, home delivery of medications to registered patients was carried out in many areas by community health workers—auxiliary nurse midwives (ANMs) and accredited social health activists (ASHA workers) (Refer for roles of different health staff in Supplementary file 1). In Kerala, the regulatory framework does not allow dispensing at HWC/SC; therefore, drugs were distributed directly from PHCs to health workers for home delivery. The drugs were dispensed for an extended period to the patients depending on the availability of drug stocks. Madhya Pradesh, Maharashtra, and Punjab states adopted an extended (2–3 months) drug dispensing at all the health facilities as per the drug availability.

Under IHCI, additional staff [Cardiovascular Health Officers (CVHOs) and Senior Treatment Supervisors (STSs)] were appointed at each project district to facilitate implementation, conduct regular supervisory visits and provide timely feedback to all stakeholders. During the lockdown period between April and May 2020, STSs and CVHOs facilitated the transport of medicines to HWCs/SCs and telephonically monitored the availability of drugs at the HWCs/SCs.

Complete reports on the number of patients who received medications through community distribution were sent by CHOs and ANMs to PHCs. Designated staff nurses at PHCs collected and compiled these reports, then sent them to the District NCD Nodal Officer. IHCI project staff compiled reports at the district level.

In addition to the health facilities report, STSs telephonically contacted a convenience sample of patients whose telephone numbers were available for verifying the availability of medications.

### 2.3. Data collection

We collected the following data for the indicators included in the analysis:

- The staff nurse reported the number of HWCs/SCs that received drugs from PHCs or district warehouses for community distribution out of the total number of functional HWCs/SCs in the district.
- Staff nurses reported to the district NCD nodal officer the number of patients who received drugs through community distribution out of total patients registered for hypertension treatment in any public sector facilities under IHCI.
- The number of patients who reported availability of drugs with them, out of total patients contacted through phone calls by the STS.

### 2.4. Data analysis

Using descriptive statistics, we calculated the proportion of HWCs/SCs implementing IHCI by the state that received antihypertensive drugs. We estimated the proportion of patients who received medicines either through home delivery or collected at the local HWC/SC per the facility records of total hypertension patients registered in any public sector health facility under IHCI until March 2020. We calculated the proportion of registered hypertensive patients who telephonically reported the availability of drugs.

## 3. Results

### 3.1 Proportion of HWCs/SCs who received antihypertensive drugs in IHCI districts for community-level distribution

**Figure 1:**
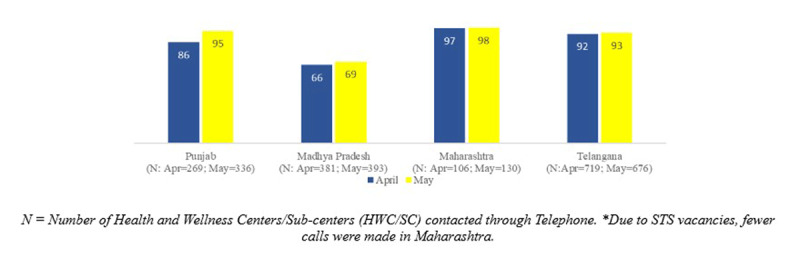
Proportion of HWC/SC which received antihypertensive drugs for community-level distribution in 29 districts, India: April (N = 1,475) & May 2020 (N = 1,535).

Of the 4,245 HWCs/SCs in the project districts, we contacted a convenience sample of 1,475 and 1,535 HWCs/SCs telephonically in four IHCI States during April and May, respectively. The contacted facilities represented approximately more than one-third of all facilities in the program. Of these facilities, 85% and 88% had received antihypertensive medications from their PHCs or district warehouse in April and May 2020, respectively (Figure 1). In Kerala, we did not monitor this indicator due to legal barriers in antihypertensive drug distribution from SCs; drugs were distributed directly from PHCs for home delivery, or patients visited the primary care health facility.

### 3.2 Received drugs as per facility reports

Among 721,675 patients registered until March 31, 2020, in any public sector health facilities, 38% (277,464) received antihypertensive drug refills based on the patient’s prior prescription either through one of the 4,245 HWCs/SCs or through home delivery of medications by frontline workers. The proportion was highest in Maharashtra (59%), followed by Telangana (52.9%). The proportion was lowest (21%) in Punjab, despite the availability of drugs in more than 80% of the facilities (Table 1).

**Table 1:** Community-level distribution of antihypertensive drugs at HWC/SC or through home delivery during COVID-19 related lockdown in 29 districts, India, April–May 2020.

Name of State (Number of districts)	Number of hypertension patients registered under IHCI till 31 March 2020	Of those registered, number of patients to whom drug delivered through a community-level distribution (23 Mar–31 May)	Percentage of registered patients provided drugs through community-level distribution

Maharashtra (4)	158,629	93,784	59.1
Telangana (10)	92,628	49,001	52.9
Kerala (4)	279,746	87,244	31.2
Madhya Pradesh (6)	93,066	26,913	29.0
Punjab (5)	97,606	20,522	21.0
Total (29)	**721,675**	**277,464**	**38.4**

### 3.3 Self-reported access to antihypertensive medications during the lockdown

**Figure 2:**
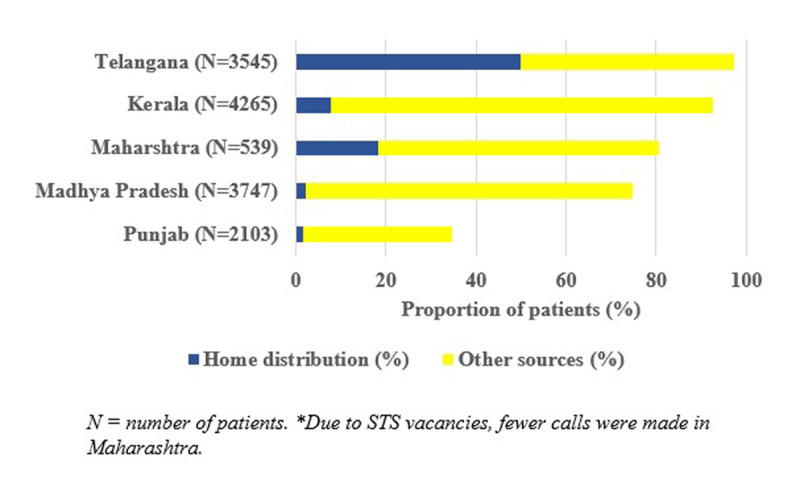
Patients’ self-reported availability of drugs assessed by telephonic calls in 29 districts, India, April–May 2020.

Overall, we telephonically contacted 16,595 patients in April and May 2020 with an 85.6% response rate. Of the 14,199 patients who responded, 11,372 (80%) had antihypertensive medications available with them. However, the proportion varied between states, the lowest being in Punjab (35%) and the highest in Telangana (97%) (Figure 2). The proportion of patients receiving antihypertensive medications through home delivery was highest in Telangana (51%), followed by Maharashtra (18%). A small proportion of patients received drugs at home in Kerala (8%), Punjab (2%), and Madhya Pradesh (2%). Overall, only 4% of patients reported having more than a one-month supply of medications, the highest being in Maharashtra (29%), followed by Punjab (11%) and Madhya Pradesh (0.3%).

## 4. Discussion and Conclusions

We demonstrated the feasibility of community-level drug distribution for hypertension patients during the COVID-19 lockdown in India. The operationalization of HWC/SC, availability of drugs at the peripheral level health facilities, and availability of health workers for outreach activities led to variable success in different states.

We anticipated that service delivery disruptions related to COVID-19 restrictions would negatively affect treatment continuity for enrolled patients. Initial data from different countries showed that patients with chronic diseases such as cardiovascular disease and diabetes were at a higher risk of becoming severely ill and dying from the COVID-19 [8,9]. Besides, disruption of hypertension and other chronic disease care could lead to an excess of acute and fatal cardiovascular disease cases and other non-COVID-19 conditions, increasing the burden on hospitals [10,11,12]. To ensure the continuation of essential health services for patients with chronic diseases during India’s COVID-19 epidemic, we adopted a mitigation strategy of decentralized, patient-centered care: community distribution of medicines through frontline health workers for patients with blood pressure under control. This adaptive response was facilitated by the Government of India’s guidelines on continuing essential health services [6].

Our data demonstrated the swift adoption of community distribution of medications and variable success in maintaining access to hypertension treatment during the COVID-19 lockdown in different states. While the uptake of multi-month refills was restricted by medication supply, other disease programs that have used the multi-month distribution of medications have demonstrated this intervention to be feasible, acceptable, and valuable to improve retention in care [13,14]. States that could rapidly shift the drug stocks to HWCs and SCs and had human resources to do outreach activities had better treatment coverage. Kerala has one of the best primary health care systems among Indian states, but the legal barriers in dispensing drugs at HWCs and SCs, which are much nearer to the patient’s home, might have influenced the coverage.

The initial success of these patient-centered adaptive strategies adopted under IHCI demonstrated the feasibility of ensuring a continuum of care to patients with hypertension whose chronic disease care was threatened with disruption in a pandemic scenario. This study’s strengths include the large sample size of facilities and patients and real-world implementation context, which suggest that the interventions are likely to be feasible at scale. Lessons learned may be replicated for other chronic diseases such as diabetes in the short term and have the potential over the long term to map the path to a more efficient, decentralized, and patient-centered model of chronic care delivery.

However, the program faced many challenges, and state health departments need to optimize these interventions. Health is a state subject in India. Despite the national guidance on extended prescriptions for patients with chronic diseases, only three of the five IHCI states had issued similar guidelines due to constraints on drug stocks’ availability. The provision of extended prescriptions needs to be supported by streamlined drug procurement and robust supply chain management systems. Intensive monitoring and rigorous follow-up by the project staff ensured adequate antihypertensive drugs to facilitate community-level distribution.

Our study had several limitations. Although pharmacists allocated a sufficient quantity of medications to health facilities based on the number of registered patients, we could not quantify the total amount of pills received at each facility and the consumption. Secondly, the study lacked comparison, non-intervention, non-IHCI districts. It was challenging to collect data as the intervention was implemented in the COVID-19 lockdown phase when staff was diverted to do COVID-19 work and documentation of NCD activities was limited. The third limitation was that we contacted a convenience sample of facilities and patients for the availability of drugs, which may have been biased.

Additionally, reports on patients having medicines on hand relied on self-reports and could not be verified at the field level. In Kerala, the self-reported availability of drugs was higher than the proportion reported by staff nurses based on the reports received from frontline workers. The discrepancy could be due to difficulty collecting reports as drugs were distributed directly from PHCs to health workers for home delivery. Due to high literacy and awareness among patients in Kerala, they might have procured drugs from private pharmacies.

We might have overestimated the availability of drugs based on calls because the STSs were more likely to call patients whose phone numbers were available and who might have been visiting the facilities regularly before COVID-19. The estimate was lower based on facility-based data regarding patients who received drugs because the denominator included all registered patients irrespective of the regularity of follow-up or availability of phone numbers.

Additional patient-level outcome data is required to define further the impact of these mitigation interventions on the successful delivery of hypertension care and treatment. Besides, a description of costs and other required healthcare resources will help future program planning during COVID-19 and beyond.

The WHO has promoted the concept of ‘Build Back Better’: leveraging the unavoidable stresses of the COVID-19 pandemic to improve health care and achieve healthier populations [10]. Once established in the health care delivery system, extended, multi-month prescription refills and community-based drugs distribution will improve patient-centered primary health care to achieve the goal of treating an additional 45 million hypertensives on the path to universal health coverage.

## Additional File

The additional file for this article can be found as follows:

10.5334/gh.1010.s1 Supplementary File 1. Qualification and Responsibilities of different health staff for IHCI/NCD management.
