# Supplementary material for: Interventions to Ensure the Continuum of Care for Hypertension During the COVID-19 Pandemic in Five Indian States—India Hypertension Control Initiative
Source: Glob Heart. 2021 Dec 8;16(1):82. doi: 10.5334/gh.1010 (PMC8663742; doi:10.5334/gh.1010)
Supplement: Supplementary File 1. — Qualification and Responsibilities of different health staff for IHCI/NCD management. [file gh-16-1-1010-s1.pdf]

Supplementary file 1:

Qualification and Responsibilities of different health staff for IHCI/NCD management

Nurse/GNM:

Qualifications: GNM (General Nursing and Midwifery) qualification as recognised by Nursing Council of India.

Job responsibilities:

- To assist Medical Officers in Management and follow-up of patients attending the NCD Clinic.
- To counsel patients and their family members about risk factors of NCDs.
- To provide home based care.
- Any other job assigned by concerned officers.

Community Health Officer (CHO):

Qualifications: Bachelor of Science (B.Sc) in Community Health or GNM or B.Sc Nursing or an Ayurveda practitioner trained and certified through IGNOU/other State Public Health/Medical Universities

Job responsibilities:

- Ensure that all households in the service areas are listed, empanelled and a database is maintained in digital format/ paper format as required by the state.
- Provide clinical care as specified in the care pathways and standard treatment guidelines for the range of services expected of the HWC.
- Clinical care provision would include coordinating for care/ case management for chronic illnesses based on the diagnosis and treatment plan made by the Medical Officer/specialists who will initiate treatment for chronic diseases, dispense drugs as per standing orders by the medical officer.
- Focus attention in screening for chronic conditions on screening, enabling suspected cases confirmed and initiating treatment based on plans made by medical officer/specialists. As a team, ensure adherence, along with counselling and support as needed for primary and secondary prevention efforts.
- Support the team of MPWs and ASHAs on their tasks, including on the job mentoring, support and supervision and undertaking the monitoring, management, reporting and administrative functions of the HWC.
- Support and supervise the collection of population based data by frontline workers, collate and analyse data for planning and report the data to the next level in an accurate and timely fashion Use HWC and population data to understand key causes of mortality, morbidity in the community and work with the team to develop a local action plan with measurable targets, including a particular focus on vulnerable communities.
- Guide and be actively engaged in community health promotion including behaviour change communication.

Auxiliary Nurse Midwife (ANM):

Qualifications: ANM qualification as recognised by Nursing Council of India.

Job responsibilities:

- Population enumeration to cover the eligible population
- Complete Community Based Assessment Checklist (CBAC) for NCD screening and identify individuals with high risk behaviours. Review completed CBAC.
- Supportive supervision through joint visits with ASHA, where required in order to motivate people to attend the screening day.
- Raising awareness about NCDs, including about the effects of tobacco consumption, alcohol use, obesity, family history, lack of exercise, unhealthy diets.
- Lifestyle counselling/ Behaviour Change Communication (BCC)
- Screening for hypertension, diabetes, and breast cancer, cervical cancer and oral cancer at the sub centre level and referring the individual who needs confirmation and initiation of treatment plan.
- Provide follow-up management for patients like monthly drug supply, periodic BP/ blood sugar measurement, referral for complication.
- Maintain village register and NCD register with relevant data.
- Co-ordinate with the PHC team such as MO, Staff nurse, Laboratory Technician and other staff, in smooth implementation of the NPCDCS Programme.

Accredited Social Health Activist (ASHA):

Qualifications: Standard 7 or above.

Job responsibilities:

- Listing of all adults above the age of 30 years
- Assisting in completion of the Community Based Assessment Checklist
- Organizing a screening day- understanding the work-flow processes
- Undertaking health promotion activity in the community
- Undertaking follow up for treatment adherence and enabling lifestyle changes
- Creating Patient Support Groups

Cardio-Vascular Health Officer (CVHO):

Qualifications: MBBS and Post-graduation in Public health

Job responsibilities:

- Accelerate implementation of hypertension treatment protocols approved by the state at the state and district levels.
- Technical support to the government at different levels and its partners for planning, training, surveillance, monitoring and evaluation of hypertension treatment and control activities.
- Technical support for extending the services for hypertension management to the community level and thereby provide patient-friendly services.
- Technical support to health facilities and community level workers in timely, accurate and complete documentation of patient blood pressure and status, as well as on aggregate reporting of hypertension data (eg, adherence to treatment, control) using a

standardized recording/reporting system, which could be paper-based and/or electronic.

- Visits to facilities at the district, sub-district and institution level to develop capacity of program staff to assess and improve the quality of service delivery based on their data.
- Regular analysis and interpretation of surveillance and epidemiological data to support appropriate follow up actions by the government and partners.
- Facilitate uninterrupted drug supply at all levels by building capacity for drug logistic management at State and district levels.
- Liaise and coordinate with national, state, and/or district level authorities, partner agencies, non-governmental organizations, health facilities, professional bodies, and others to strengthen programmatic activities, enhance political and administrative commitment.
- Build capacity of the government at different levels in training including preparation of a training plan and training of trainers, district level trainings etc.

Senior Treatment Supervisor (STS):

Qualifications: Essential with Graduation in science and Desired Post-graduation in Public health

Job responsibilities:

- Support in ensuring timely reporting from district and sub-district levels on IHMI implementation and ensure data quality.
- Provide training and technical support to healthcare workers and other stakeholders in timely, accurate and complete documentation of patient blood pressure and using a standardized recording and reporting system.
- Provide training to health care workers and others or assist the CVHO in their training.
- To conduct technical visits to facilities to develop capacity of health staff and to assess, improve the quality of service delivery based on their data while documenting good practices.
- Conduct regular analysis of surveillance and epidemiological data and share findings with supervisor to facilitate appropriate follow up.
- Help monitor drug availability and timeliness of drug delivery to health facilities.
- Undertake other technical assistance activities as deemed appropriate and necessary by the CVHO.
